# A Cross-Sectional Content Analysis Exploring Women’s Experiences of Family Support Towards Tandem Breastfeeding in a Global Facebook Group sample

**DOI:** 10.1007/s10995-026-04226-7

**Published:** 2026-01-27

**Authors:** Jessica Eve Jackson, Jenny Hallam

**Affiliations:** 1 Child, Maternal and Families Health Research, School of Health Sciences, University of Nottingham, Nottingham, UK; 2 College of Health, Psychology and Social Care, University of Derby, Derby, UK

**Keywords:** Breastfeeding, Tandem breastfeeding, Family support, Child health, Maternal health

## Abstract

**Objectives:**

This study explored how tandem-feeding mothers felt supported or unsupported by their family members, to identify common responses to this breastfeeding practice.

**Methods:**

Free-text data were collected via an online questionnaire from a global sample of 1,209 tandem-feeding mothers. An emergent content analysis was conducted on 795 statements describing supportive experiences and 1,342 statements describing unsupportive experiences. Coding categories were developed inductively from the data.

**Results:**

Five coding categories emerged from supportive comments and seven from unsupportive comments. The most prevalent supportive categories were normalisation of breastfeeding and respect for the mother’s choice (52%), emotional support and encouragement (19%), and support provided without specific context (11%). The most prevalent unsupportive categories were questioning, challenging or pressuring mothers to stop tandem feeding (34%); disapproving comments, gestures or insults that caused mothers to feel uncomfortable (22%); and misinformed comments about tandem feeding (18%).

**Conclusions for Practice:**

Tandem-feeding mothers frequently experience both support and opposition from family members, with unsupportive responses often characterised by judgment and misinformation. These findings highlight the need for increased education and visibility of tandem feeding to normalise this practice and reduce stigma. Improving family understanding of tandem feeding may enhance social support for mothers and contribute to more positive breastfeeding experiences.

**Supplementary Information:**

The online version contains supplementary material available at 10.1007/s10995-026-04226-7.

## Significance

What is already known: We know that family support can act as a positive enabler for breastfeeding. However, there is little research exploring experiences of family support specifically for women tandem breastfeeding. What this study adds: This study provides a perspective on family support for women of global Facebook breastfeeding support group. It identifies common supportive and unsupportive behaviours experienced with family members towards their tandem breastfeeding practice. Findings highlight both encouragement and opposition, demonstrating that wider normalisation and education are crucial for maternal wellbeing. Healthcare professionals can help reduce stigma, improve family support, and promote positive a breastfeeding experience.

**Supplementary Information:**

The online version contains supplementary material available at 10.1007/s10995-026-04226-7.

## Introduction

Tandem breastfeeding is defined as the continuation of feeding through subsequent pregnancies, births or adoptions of two or more children (Bronwyn, 2018). In these circumstances, the human milk’s nutritional requirements adapt for both the newborn and older children (Wesolowska et al., 2019). Tandem breastfeeding increases maternal nutritional demands, and anthropological and clinical literature highlights that closely spaced pregnancies can pose risks to maternal and infant health if maternal nutrition is inadequate (Trevathan, 2010; Cadwell & Turner-Maffei, 2021). While these sources provide a cautionary perspective, there is currently no direct empirical evidence that tandem breastfeeding itself inherently jeopardises maternal or fetal health in well-nourished populations. However, there is evidence which highlights key benefits. For example, tandem breastfeeding has been shown to nurture a sibling relationship (López-Fernández et al., 2023). Supporting tandem breastfeeding would foster optimal health benefits by encouraging the continuation of breastfeeding with safe and adequate complementary foods into the second year of an infant’s life (WHO, 2023). In a small-scale study with 15 tandem breastfeeding women in Madrid, motivations for this practice included freedom of choice and a desire for the life experience tandem breastfeeding would bring (Rodríguez Vázquez et al., 2023). Günaydın et al. (2024) conducted a study in Turkey with women who shared unique experiences of tandem breastfeeding on their child’s health, pregnancy, and breast milk. However, Erdoğan and Turan (2023) conducted a case-control study also in Turkey and identified an increased intense perception of milk deficiency for women continuing to breastfeed while pregnant. The global prevalence of tandem breastfeeding is unknown though it is not thought to be common, and there is little research exploring healthcare needs and experiences (O’rourke & Spatz, 2019).

Aker et al. (2023) conducted a phenomenological study with nine women who were tandem breastfeeding and four women breastfeeding through pregnancy. They highlighted that although there was no evidence in the literature regarding the harms of tandem breastfeeding, the women encountered many unsolicited concerns from others. This highlighted the need for support from their wider family to cope with these experienced challenges. This aligns with wider literature exploring the impact of family support on breastfeeding. For example, Karmacharya et al. (2017) identified that grandmothers act as positive enablers of breastfeeding if they have breastfeeding knowledge and firsthand experience. Additionally, there is recognition that despite breastfeeding being a biological norm it is a practice learnt through observing others (Scelza & Hinde, 2019). Where breastfeeding knowledge and practices are not present in a family culture, it has been observed to impact negatively on women’s breastfeeding choices (Alianmoghaddam et al., 2018). Therefore, it is important this study aimed to understand the experiences of both supportive and unsupported wider family support specifically for those tandem breastfeeding.

## Method

This study follows the COREQ (Consolidated Criteria for Reporting Qualitative Research) checklist to guide reporting of qualitative methods and findings. Data was collected using an online questionnaire that was shared with breastfeeding mothers via Facebook breastfeeding support groups. Once permission had been granted from the support group moderators a recruitment call, which invited mothers to participate in the study and share the link within their networks, was posted in breastfeeding support groups between June to May 2019. This purposeful recruitment strategy with snowballing elements was chosen as it is effective in generating a large cross-sectional sample. The questionnaire invited the mothers to share demographic information such as country of residence, age, identified ethnic and religious group, and the number of children (see Table 1). It also included a free-text question that enabled the mothers to share their positive and negative experiences with partners, family, and peers. This paper explores the experiences of the 1,209 women who were tandem breastfeeding at the time of data collection and responded to the free text questions that invited them to share how they had been supported and not supported by family members in their breastfeeding continuation. The experiences of partner support are explored elsewhere (Authors, under review).

The study received full ethical approval from the university college ethics committee. After clicking the link to register interest in the study the mothers received a downloadable information sheet that provided information about the study and what participation involved. After reading this information, all participants signed an informed consent form before completing the questionnaire. To minimise the risk of automated or fraudulent responses, we employed standard bot-mitigation measures and response patterns. This included a review of completion time and internal consistency during data cleaning. No responses demonstrated characteristics suggestive of automated or bot-generated entries. Each participant generated a unique participation code for anonymous and secure data storage. The code could also be used by participants to withdraw from the study, without any reason or justification, up to 14 days after taking part. No participants chose to withdraw.

**Table 1:** Participant demographics

	*N*
Geographic location	
Australia & Oceania	103
United Kingdom	448
Rest of Europe	89
Middle East & Asia	25
America & Caribbean	473
Unknown	5
Age	
18–24	49
25–34	709
35–44	444
45–54	10
Ethnicity	
Asian	42
Mixed Heritage	57
Not Stated	10
White	1067
Other	31
Religion	
Christian*	549
No religion	605
Other	54
Number of children	
2 children	909
3 or more	303

*(Including Church of England, Catholic, Protestant and all other Christian denominations)

### Data Analysis

Each of the responses was read and separated into supportive and unsupportive comments. There were participants who gave both supportive and unsupportive experiences. Once separated each comment was then reviewed and if needed the comment was separated into its parts e.g. ‘my mum openly disagrees with my breastfeeding of my 2.5 and 5-year-olds and will tell them in front of me that they are too old in an attempt to shame them into stopping’ addresses two issues - the first relates to the mother being disapproving and the second concerns the mother directly addressing the children. Consequently, this comment was split into two statements for coding. In total, there were 1,342 (63%) unsupportive statements and 795 (37%) supportive statements.

Due to the exploratory nature of the study and the large volume of data generated an emergent content analysis was used to code all the statements (Krippendorff, 2018). This approach enabled the creation of the coding manual to be guided by the insights provided by the mothers rather than the researchers’ preconceived ideas (Erlingsson & Brysiewicz, 2017). To generate the coding manual 100 unsupportive statements and 80 supportive statements were independently reviewed by each author to generate themes. The researchers then met to discuss the themes they had identified and used them to create 2 coding manuals – one for the supportive comments and one for the unsupportive comments. The coding manuals were then used to code 60 supportive and unsupportive statements with the researchers meeting after coding 20, 40 and 60 statements to discuss disagreements. During this process, two coding manuals, shown in Tables 2 and 3 were defined and agreed upon.

**Table 2:** Coding manual used for unsupportive comments

Code	Description
Disapproving comments, gestures, insults and making the mother feel uncomfortable	Statements related to disapproval such as *‘just little snide comments here and there’* were coded into this category along with statements that relayed disproving gestures ‘*family in law roll their eyes’.* Direct insults to the mother ‘*They told me that I’m* crazy’ and jokes made at the mother’s expense *‘My younger sisters can be the worst even sending me offensive memes of an adult male asking his old mother for ‘bitties’ and laughing at me’* where also coded in this category. Finally, statements that indicated that the mother was made to feel uncomfortable ‘*feel mostly uncomfortable to feed in front of them once kids were over 1*’ were coded into this category.
Questioning, Challenging and Demanding for the mother to stop	Questions around the mother’s breastfeeding such as ‘*asking for how long I am planning to breastfeed’* were coded into this category along with prompts to stop breastfeeding and ‘*telling me it’s time to wean’.* Challenges to the mother about breastfeeding centred on the child’s age *‘she is getting too old to breastfeed’*, and the child’s inability to stop ‘*he would continue to first grade (too long)*’ were coded into this category.
Disgust, sexualisation of breastfeeding, and the need to be discrete	Statements relating to disgust towards breastfeeding such as *‘they think it ‘yuck’ after 1-year-old’* as well as statements that were aligned breaching social norms ‘*I think now my eldest is over 3 and is still feeding they feel it is a little strange’* and statement that related to the person’s surprise *‘comments like are you STILL feeding*’ were coded into this category. Statements which sexualised breastfeeding ‘*There have been comments like have you got another one for me? (Whilst tandem feeding to sleep one night)*’ and exposure of breasts ‘*they were awkward initially about my breasts being on show*’ or ‘*walked out of room while I was feeding*’ were also coded into this category. Finally, statements surrounding discretion when breastfeeding such as ‘*I do sometimes get comments about being more discrete*’ and ‘*telling me to cover up’* were coded into this category.
Misled comments	Statements within this category related to misled health concerns relating to pregnancy ‘*That I needed to stop feeding my toddler because I was pregnant*’ and the newborn baby ‘*My mother-in-law said I don’t have enough milk for baby*’. Misled opinions relating to the recommended length of breastfeeding ‘*they don’t think it’s necessary past 6 months’* and the nutrition benefits ‘*there is no benefit after a certain age*’ were also included in this category. Misled beliefs about the wider impact that breastfeeding had such as ‘*they also blame any behavioural issue that comes up on breastfeeding*’ and *‘they are too clingy to me*’ were also included in this category. Finally, in reference to the mother abusing her child(ren) ‘*my extended older members of the family have threatened to report me to social services’* and pro formula beliefs ‘*in-laws were formula feeders and say the formula is easier and just as good*’ were coded into this category.
Directly addresses child	Statements that indicated that the child was directly addressed and shamed for breastfeeding were included in this category ‘*telling my child that she is a big girl and doesn’t need milkies’* as well as statements in which the child was encouraged to drink an alternative ‘*offers him cows milk instead.*’
Does not disclose/no contact with family	Statements in which the mother did not disclose breastfeeding ‘*I’ve generally stopped telling people that he still has a nighttime feed to go to sleep*’ or discuss breastfeeding ‘*I cut her off or change the subject. We usually avoid mentioning it to keep the peace’* were coded into this category. Statements that indicated that there was no contact with family ‘*I don’t have contact with my family*’ were also included.

**Table 3:** Coding manual used for supportive comments

Category	Description
Practical support and support with feeding.	Statements were coded into this category if they related to looking after the mother’s physical needs ‘*always get food and water for me and a seat as soon as I come in’* or taking action to lighten the mother’s load ‘*Mum came over to help me out. The first few weeks are hard when establishing feeding and your supply especially if you have other children’*. Statements that referenced actions taken to make feeding easier ‘*When we visit*,* my mum arranges the beds so we can continue extended breastfeeding’* were also coded into this category. Finally, statements relating to helping to establish feeding *‘mom helped get started’* or facilitating feeding *‘supports feeding breastmilk while I work’* were coded into this category.
Emotional support and encouragement for the mother.	Statements were coded into this category if they related to encouraging comments like ‘*my mum especially has said that she thinks it’s amazing*’ or recognising the mother’s achievement ‘*some say it’s so good you have fed for this long*’. Statements that related to expressing pride ‘*proudly tell everyone about my breastfeeding achievements*’ and encouragement ‘*my in-laws encourage me to continue’* were also coded into this category.
Recognising the health benefits and being educated about tandem breastfeeding.	Statements were coded into this category if they related to understanding the health benefits of breastfeeding ‘*they really believe my kid’s good health is derived from BF’* as well as the emotional benefits of breastfeeding ‘*they also think that it’s really sweet how much he relaxes and enjoys the moment’*. Statements were also coded into this category if they referred to the family members actively engaging with educational materials ‘*My mother-in-law shows me articles about how good it is’*
Breastfeeding is normalised and the mother’s choice is respected.	Statements in which family members accepted breastfeeding ‘*Positivity by treating bf as normal’* were coded into this category along with statements in which the mother had a family role model ‘*my aunt also tandem fed her first 2 kids and breastfed her 3rd u til 4.5 so it is quite accepted*,* I feel comfortable feeding around them’.* Statements which referenced a lack of negative reaction were also coded into this category *‘Now 4yrs 7mth later and another baby added*,* they don’t even bat an eyelid’* Finally, statements that related to respecting the mother’s decision ‘*They don’t question my decision to bf or see anything unusual in it*’ were coded into this category along with statements that indicated that the mother was not challenged ‘*They’ve mostly left me to it*.’
Support with no context.	Statements such as ‘*Very supportive’* were coded into this category because they suggested support but did not give any detail as to the kind of support received.

To test inter-rater reliability 20% of the unsupportive and supportive comments were independently coded by the two authors using the agreed coding manuals. A Kappa measure of agreement was then performed. The kappa value was 0.81, *p* <.001 for the *Supportive Statements* and 0.638, *p* <.001 for *Unsupportive Statements*. Values over 0.5 indicate a good agreement and so the second author used the manuals to code the whole data set (Pallant, 2016).

## Results

The frequency and percentage coded category for the total 1,342 (63%) unsupportive statements and 795 (37%) supportive statements are outlined in Table 4.

**Table 4:** Frequency of coded categories

	*N (%)*
Unsupportive category	
Questioning, challenging, and demanding for the mother to stop	451 (34)
Disapproving, gestures, insults and making the mother feel uncomfortable	290 (22)
Misled comments	248 (18)
Disgust, sexualisation of breastfeeding, and the need to be discrete	219 (16)
Directly addresses child	79 (6)
Does not disclose/no contact with family	55 (4)
Supportive category	
Breastfeeding is normalised and the mother’s choice is respected	411 (52)
Emotional support and encourages the mother	155 (19)
Support with no context	88 (11)
Practical support and support with feeding	69 (9)
Recognising the health benefits and being educated about tandem breastfeeding	72 (9)

## Discussion

This content analysis has indicated that women practicing tandem breastfeeding are more likely to receive unsupported comments from their family members than supportive ones. Snyder et al. (2021) identified a lack of familial support as a barrier to breastfeeding and therefore our findings indicate that this is the case for tandem breastfeeding women. Our findings add new knowledge specifically for this sample of underrepresented women within breastfeeding research.

### Unsupportive Comments

The most frequent unsupported category comments were *Questioning*,* challenging*,* and demanding for the mother to stop* (*n = 451*,* % = 34).* Therefore, the results indicate that it was common for these women to experience a family member disputing their continued breastfeeding practices and making it explicitly clear that they viewed it as a behaviour that the women should end immediately. Wider literature, exploring the experiences of women who continue to breastfeed past infancy, has also highlighted that as a breastfed infant gets older women can experience an increase in interrogative comments from others enquiring when they intend to stop (Jackson & Hallam, 2021). Therefore, the social pressure on a breastfeeding duration limit should be highlighted when supporting women in their decision to tandem breastfeed and help them build resilience for responding to others who may question their practice.

The second most frequent unsupportive comments were *Disapproving comments*,* gestures*,* insults and making the mother feel uncomfortable* (*n = 290*,* % = 22).* These results indicate that tandem breastfeeding women would experience being shamed by a family member because of their practices. It is evident here that breastfeeding social stigma was present within these women’s family networks. Therefore, tandem breastfeeding women would benefit from additional support to understand how to address their positionality and overcome challenging family social dynamics.

The next most frequent unsupportive category comments were *Misled comments* (*n = 248*,* % = 18).* This indicates that women frequently experienced a family member offering breastfeeding advice which was not based on current evidence-based guidance. This aligns with Aker et al. (2023) phenomenological study the women encountered many unsolicited concerns from others regarding the harms of tandem breastfeeding. It also aligns with Chang et al. (2021) who conducted a qualitative systematic review to examine partners’ and family members’ views and experiences of supporting breastfeeding. It highlighted that families located in different global geographical can have incorrect understandings of breastmilk and normal breastfeeding practices. This indicates a need to tailor family-centred education interventions in local communities. These results also align with Alianmoghaddam et al. (2018) who highlighted that when knowledge and practices are not present in a family culture, it has been observed to have a negative impact on breastfeeding women. Gharaei et al. (2020) found positive breastfeeding outcomes in the prenatal and postnatal period where maternal grandmothers attended family-centred breastfeeding education programs. However, further design, implementation, and evaluation of family-centred education programs specifically for the promotion of tandem breastfeeding is needed.

The next similarly frequent unsupportive category comments were *Disgust*,* sexualisation of breastfeeding*,* and the need to be discrete* (*n = 219*,* % = 16).* These indicate that women often find themselves having to breastfeed in situations where their family would openly shame and prohibit them as socially abnormal, unacceptable, deviant or perverted. This aligns with wider literature highlighting the social stigma when breastfeeding in public where women are seen as disrespectful exhibitionists performing illegal body exposure (Bresnahan et al., 2019). In these circumstances, the acceptance of public breastfeeding is dependent on mothers displaying discretion. Kent et al. (2023) have highlighted that to address this communities need to challenge this discourse and problematise the relevance of discretion.

### Supportive Comments

The most frequently supported category comments were *Breastfeeding is normalised and the mother’s choice is respected* (*n = 411*,* % = 52).* This indicates the importance of normalising tandem breastfeeding practices. Thorley (2019) has explored the perception of breastfeeding normality and highlighted where a culture of breastfeeding is in decline, women are unlikely to view it as the normal way to feed their infant. Our results also reinforce what Karmacharya et al. (2017) identified, that family members with an understanding of breastfeeding behaviours and firsthand experience can act as positive enablers. The view that it is the mother’s choice aligns with research which has highlighted that breastfeeding is predominantly seen as a woman’s decision and that gendered family roles are a large cultural factor in this (Chang et al., 2021). This was also seen in a qualitative study conducted by Haigh (2020) which aimed to explore young mothers’ experiences of breastfeeding whilst living with their family. It demonstrated a woman’s need for autonomy in their breastfeeding decisions regardless of the opinions and views of others around them but where breastfeeding is normalised within the family, women also experience higher levels of support. This again highlights the importance of family-centred interventions but specifically explores and addresses the impact of generational experiences of breastfeeding practices.

The next frequent supportive category comments were *Emotional support and encourage the mother* (*n = 155*,* % = 19).* This indicates that these women, who have established tandem breastfeeding, experience emotional support from their family members. Jackson and Hallam (2020) conducted a qualitative exploration of women’s experiences of breastfeeding beyond infancy, which highlighted that where emotional support for breastfeeding was not received within their families and local communities, women joined online international communities for emotional support which helped validate their breastfeeding practices. However, Orchard and Nicholls (2022) conducted a systematic review which specifically explored the impact of online social media breastfeeding support and stated their success is dependent on specific content shared, individual contributors, and community dynamics. This again highlights the need for tailored interventions designed to help families better communicate and deduce what support tandem breastfeeding women need, whilst reflecting on the dynamics of different family relationships.

## Conclusion

This content analysis has shown that women who tandem breastfed disproportionally experience unsupported comments from their family members. While online recruitment via Facebook support groups and self-reported data have inherent limitations, this approach enabled access to a geographically diverse sample of mothers practising the relatively rare behaviour of tandem breastfeeding. Recruitment via Facebook support groups likely introduced selection bias, as participants were already engaged in breastfeeding–friendly online communities. Consequently, the perspectives captured may not reflect the experiences of mothers who are less connected to online networks or who lack access to peer support. Additionally, the categorisation of comments as “supportive” or “unsupportive,” while guided by an established deductive content analysis approach, represents a simplification of the complex spectrum of social responses and may not capture more nuanced or context-dependent attitudes towards tandem breastfeeding. We recognise that more nuanced gradations should be explored in future research using different methodological approaches to build a more comprehensive body of evidence on this topic. However, these findings add new knowledge specifically for this sample of women who are underrepresented within wider breastfeeding research.

Given that the prevalence and impact of supportive and unsupportive comments regarding tandem breastfeeding remain under-researched, future studies should examine whether such comments are associated with changes in tandem breastfeeding practices, and whether increasing supportive comments or reducing unsupportive comments influences the frequency or quality of tandem breastfeeding, thereby providing an evidence base for broader community-level interventions. Future research should also consider maternal well-being in greater depth, including practical challenges associated with tandem breastfeeding, explore whether mothers have discussed their feeding practices with healthcare or lactation professionals, and examine how experiences of tandem breastfeeding may vary according to child age and cultural or national context. Lastly, we did not examine the duration or exclusivity of breastfeeding for either child, which represents an important limitation. Future studies should report paired data on child ages and feeding patterns to advance understanding of tandem breastfeeding.

The findings underscore the global relevance of health education and family-centred interventions that support tandem breastfeeding in culturally diverse settings. They indicate that tailored family-centred interventions are needed to support women to continue breastfeeding through pregnancy and maintain tandem breastfeeding. These interventions need to consider the impact of social pressures on a breastfeeding duration limit. They need to promote education about the benefits of tandem breastfeeding and challenge the current discourse of discretion. They should also specifically explore and address the impact of generational experiences of breastfeeding practices. Health promotion and family-centred interventions should also consider local norms, family structures, and sources of social support, ensuring guidance is non-judgemental and culturally sensitive. Women will benefit from additional interventions to help them understand their specific needs, how to address their positionality as a breastfeeding woman and how to overcome challenging family social dynamics. The study included a sample of women who had successfully established tandem breastfeeding. Further research should explore challenges for those who were not able to overcome their challenges to determine more reliable predictors.

Finally, although participants were recruited globally, the broad regional categories encompass highly heterogeneous populations in terms of culture, socioeconomic status, and access to healthcare. Additionally, the sample was predominantly white, largely non-religious, and recruited via international Facebook breastfeeding groups, and therefore does not represent the global population of mothers. Nor did we have sufficient numbers to compare mothers by religion, limiting subgroup analyses. Consequently, findings should be interpreted as exploratory and indicative of general patterns rather than representative of specific countries or subpopulations. It is also important to note that this study was limited to women. However, it is acknowledged that non-binary and trans* persons can also breastfeed or chestfeed. However, research exploring the experiences of practices for these parents is limited (Jackson et al., 2023). Therefore, it is important to expand this body of evidence to obtain a more inclusive sample.

## Supplementary Information

Below is the link to the electronic supplementary material.

Supplementary material 1 (DOCX 13.4 kb)

## Data Availability

The data is available directly from the author as reasonable request.
