# Supplementary material for: A Cross-Sectional Content Analysis Exploring Women’s Experiences of Family Support Towards Tandem Breastfeeding in a Global Facebook Group sample
Source: Matern Child Health J. 2026 Jan 27;30(3):377–84. doi: 10.1007/s10995-026-04226-7 (PMC13038786; doi:10.1007/s10995-026-04226-7)
Supplement: Supplementary file 1 — Supplementary material 1 (DOCX 13.4 kb) [file 10995_2026_4226_MOESM1_ESM.docx]

| **COREQ Domain checklist** | | **Description** |
| --- | --- | --- |
| **Research Team & Reflexivity** | 1. Interviewer/facilitator | Open-text responses were collected using an anonymous online survey |
|  | 2. Credentials | Researchers hold PhD in Public Health and Psychology. |
|  | 3. Occupation | Associate Professor and Senior Lecturer |
|  | 4. Gender | Women |
|  | 5. Experience & training | Trained in qualitative research. |
|  | 6. Relationship with participants | Participants were recruited via Facebook support groups. |
| **Study Design** | 7. Methodological orientation | Deductive content analysis guided by prior frameworks; thematic coding applied. |
|  | 8. Sampling | Convenience sampling via international Facebook breastfeeding groups. |
|  | 9. Method of approach | Online survey link posted in breastfeeding support groups. |
|  | 10. Sample size | N = [number] participants who provided qualitative responses. |
|  | 11. Non-participation | N/A |
|  | 12. Setting of data collection | Online survey accessible globally |
|  | 13. Presence of non-participants | N/A survey only |
|  | 14. Description of sample | Demographics presented |
| **Data Collection** | 15. Interview guide | Open-ended survey questions |
|  | 16. Repeat interviews | N/A single survey response per participant. |
|  | 17. Audio/visual recording | N/A survey text only. |
|  | 18. Field notes | Notes made during data cleaning and coding |
|  | 19. Duration | N/A survey completion time checked for consistency |
|  | 20. Data saturation | Saturation was achieved as no new subthemes emerged after coding the final responses. |
|  | 21. Transcripts returned | Not applicable – online survey. |
| **Data Analysis and Findings** | 22. Number of coders | Two independent researchers coded the data. |
|  | 23. Description of coding | Deductive coding based on pre-defined supportive / unsupportive categories |
|  | 24. Software | NVivo/Excel used for data organisation and coding. |
|  | 25. Participant checking | Participants were not re-contacted |
|  | 26. Derivation of themes | Themes derived from deductive categories . |
|  | 27. Quotations presented | Representative participant quotes included. |
|  | 28. Data and findings consistent | Themes & illustrative quotes demonstrate consistency. |
|  | 29. Clarity of major themes | Major themes are explicitly described. |
|  | 30. Clarity of minor themes | Minor subthemes and nuances are presented in the discussion. |
